# Personal

**Published:** 1894-03

**Authors:** 


### Personal.

Dr. William Thomas Coggin, of Athens, Ga., performed the first American symphyseotomy in Freedman, Ala., March 12, 1892. Dr. Coggin, in Heidelberg in 1890, heard of Morrisani's success and then decided to prefer symphyseotomy to craniotomy whenever a case should come under his care in which one or the other alternative should be presented. Dr. Coggin operated upon the wife of a miner, who had been fifteen and a half hours in labor and who had a contracted pelvis of the justo-minor type, computed to be one-third smaller than the average. After opening the symphysis, he applied the forceps and delivered a male fetus, weighing eleven and three-quarter pounds. It was noted that the pubic bones became separated two and three-quarter inches. There was no injury produced by the extraction of the child either to the sacro-iliac synchondrosis or to the soft parts. Under a proper restraining apparatus the pubes readily united, there was no lameness, and the mother and infant both did well. This information is obtained from Dr. Harris's paper in the *Annals of Gynecology and Pediatry* for February, 1894, and we regret that we did not possess these facts to incorporate in our editorial on symphyseotomy in the February issue of the JOURNAL.

---

DR. P. O. HOOPER, for many years Superintendent of the Arkansas State Lunatic Asylum, resigned to take effect December 1, 1893. The *Arkansas Gazette* regards Dr. Hooper's retirement as due to political influences. Whatever cause may have led to this result is a matter of deep regret to Dr. Hooper's professional friends throughout the country, as well as to those persons more immediately concerned in the conduct of the asylum, that this step was thought necessary. It will be difficult to find a successor who is as well fitted for the charge as the distinguished Superintendent who has retired.

---

DR. J. HENRY CARSTENS, of Detroit, has removed his office and residence from 21 Macomb street to 620 Woodward avenue. His practice is entirely restricted to gynecology, abdominal surgery and consultations. His hours are 8 to 9 A. M. and 1 to 3 P. M.

---

DR. R. HARVEY REED has removed from Mansfield to Columbus, O. His address is 150 Broad street.

---

DRS. EDWIN WALKER and A. M. OWEN, of Evansville, Ind., have established a sanitarium in that city, the object of which is to provide a suitable place for the care of medical and surgical cases. Those who are acquainted with these distinguished physicians will readily understand that success in their enterprise is already assured. Patients can make no mistake in placing themselves under the roof of the Evansville Sanitarium for treatment.

---

DR. MONTGOMERY A. CROCKETT, of 452 Franklin street, Buffalo, announces that his practice will hereafter be confined to gynecology and obstetrics. His hours are 1 to 3 P. M. Dr. Crockett published an interesting article in the February issue of this magazine, entitled, Observations on the Results of Removal of Diseased Uterine Appendages, which will well repay careful study.

